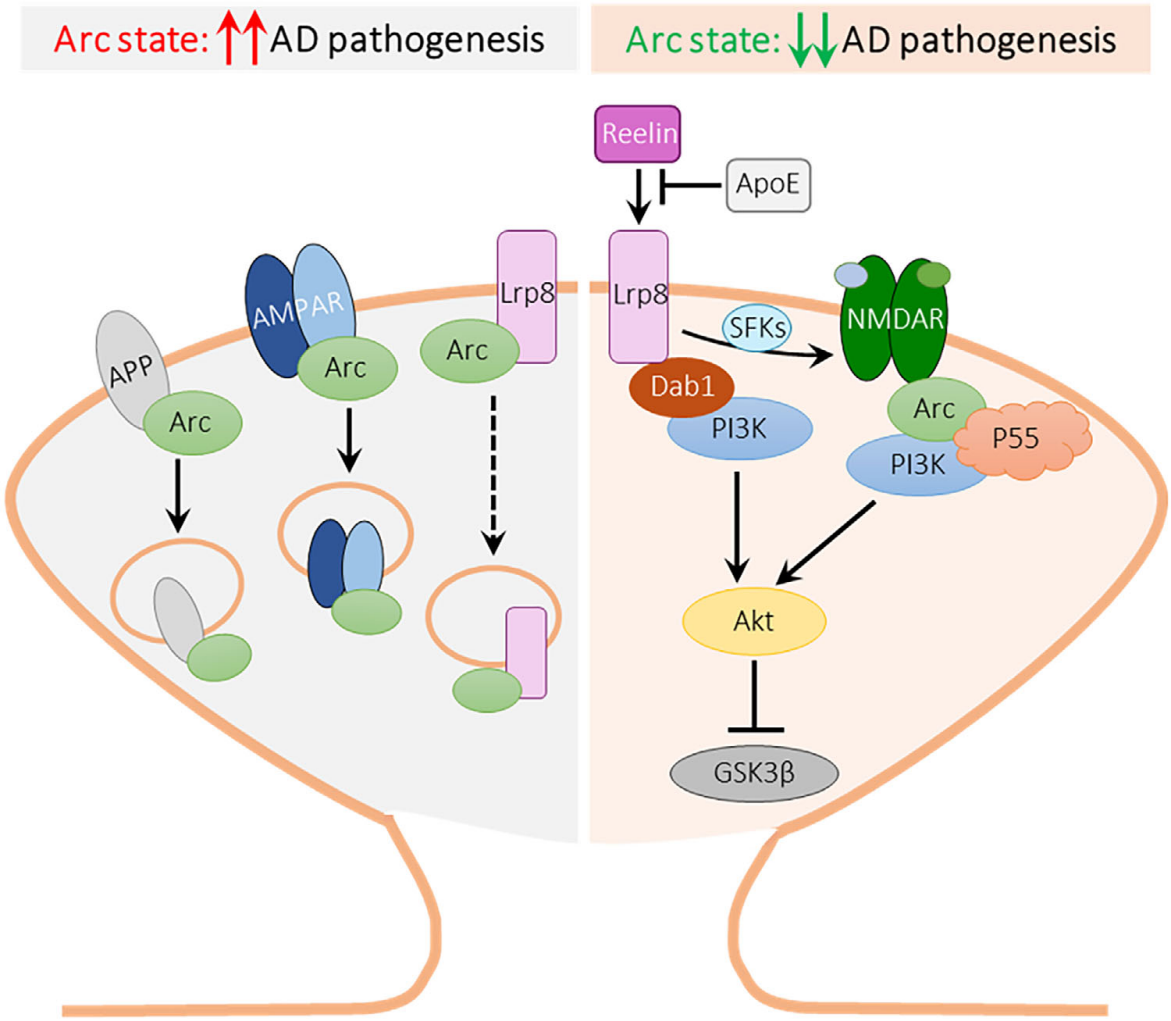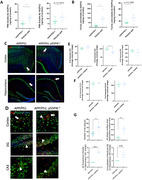# Disruption of Arc‐NMDAR‐p55PIK‐AKT pathway increases Aß42 and Aβ plaque deposition, and reduces CA3 neuron counts in APPswe/PS1∆E9 mouse model

**DOI:** 10.1002/alz.095521

**Published:** 2025-01-09

**Authors:** Luis Gladulich, Camila Cuoco, Paul F Worley

**Affiliations:** ^1^ Johns Hopkins University School of Medicine, Baltimore, MD USA

## Abstract

**Background:**

Arc is a synaptic immediate early gene that mediates two distinct pathways at excitatory synapses. Canonically, Arc accelerates endocytosis of AMPA receptors by direct binding to TARPgs and endocytic machinery and thereby contributes to mGluR‐LTD. Arc also acts at recently potentiated synapses, where it is phosphorylated by CaMKII and binds NMDAR subunits NR2A and NR2B and recruits the PI3K adaptor p55PIK to assemble a signaling complex that activates AKT and inhibits GSK3β. This Arc‐NMDAR‐p55PIK‐AKT signaling mediates metaplasticity that prevents depotentiation of recently potentiated synapses and is important for memory updating (Arc‐LTP pathway). Arc‐NMDAR‐p55PIK‐AKT signaling contributes significantly to total AKT activity, which is a pro‐growth/pro‐survival signal, and can be selectively disrupted by conditional deletion of p55PIK. The Arc‐LTD pathway has previously been shown to enhance APP processing to generate Aß by recruiting g‐secretase to endosomes. Here, we examine the role of the Arc‐LTP pathway in Aß amyloidosis, and cell viability using the APPswe/PS1∆E9 mouse model.

**Method:**

We bred p55PIK floxed(p55PIK_flox/flox_) mice with APPswe/PS1∆E9(APP/PS1) mice and Nestin‐Cre to generate APP/PS1+ mice that were also conditional knock‐outs for p55PIK. p55PIK_flox/flox_; APP/PS1+ mice without Cre were examined as controls. Mice were examined at 6 m. Aβ40/42 were measured via ELISA and IF staining. Cell count was done via NeuN co‐staining. Comparisons used Prism 10 software and student’s T‐test for comparison and considered statistically significant if p<0.05.

**Result:**

Compared to APP/PS1, APP/PS1;p55PIK KO mouse forebrain exhibited roughly two‐fold increase in Aβ42 in both the PBS and formic‐acid soluble fractions and a corresponding increase in both number and size of Aβ plaques. Aß40 levels were not significantly increased. Neuronal cell counts in the hippocampus suggested ∼20% reduction in the CA3 region without changes in other regions.

**Conclusion:**

Preliminary studies suggest a role for Arc‐NMDAR‐p55PIK‐AKT signaling in APP processing. The prominent increase of Aß42 and plaque area are consistent with a role in effective g‐secretase processing of APP while the reduction of CA3 cell counts suggests a role in neuron viability. Arc acting at potentiated synapses appears to have a protective effect. Ongoing studies are examining larger cohorts of both genders at 6m and 12m